# The Importance of Poly(ethylene glycol) and Lipid Structure in Targeted Gene Delivery to Lymph Nodes by Lipid Nanoparticles

**DOI:** 10.3390/pharmaceutics12111068

**Published:** 2020-11-09

**Authors:** Danijela Zukancic, Estelle J. A. Suys, Emily H. Pilkington, Azizah Algarni, Hareth Al-Wassiti, Nghia P. Truong

**Affiliations:** 1 Department of Drug Delivery, Disposition and Dynamics, Monash Institute of Pharmaceutical Sciences, Monash University, Melbourne, VIC 3052, Australia; dzuk0001@student.monash.edu (D.Z.); Emily.Pilkington1@monash.edu (E.H.P.); azizah.algarni@monash.edu (A.A.); harry.al-wassiti@monash.edu (H.A.-W.); 2 Department of Microbiology and Immunology, Peter Doherty Institute for Infection and Immunity, University of Melbourne, Melbourne, VIC 3052, Australia

**Keywords:** gene delivery, plasmid DNA (pDNA), transfection, lymph node targeting, PEGylation, lipid nanoparticles, tween, nanomedicine, vaccine, immunotherapy

## Abstract

Targeted delivery of nucleic acids to lymph nodes is critical for the development of effective vaccines and immunotherapies. However, it remains challenging to achieve selective lymph node delivery. Current gene delivery systems target mainly to the liver and typically exhibit off-target transfection at various tissues. Here we report novel lipid nanoparticles (LNPs) that can deliver plasmid DNA (pDNA) to a draining lymph node, thereby significantly enhancing transfection at this target organ, and substantially reducing gene expression at the intramuscular injection site (muscle). In particular, we discovered that LNPs stabilized by 3% Tween 20, a surfactant with a branched poly(ethylene glycol) (PEG) chain linking to a short lipid tail, achieved highly specific transfection at the lymph node. This was in contrast to conventional LNPs stabilized with a linear PEG chain and two saturated lipid tails (PEG-DSPE) that predominately transfected at the injection site (muscle). Interestingly, replacing Tween 20 with Tween 80, which has a longer unsaturated lipid tail, led to a much lower transfection efficiency. Our work demonstrates the importance of PEGylation in selective organ targeting of nanoparticles, provides new insights into the structure–property relationship of LNPs, and offers a novel, simple, and practical PEGylation technology to prepare the next generation of safe and effective vaccines against viruses or tumours.

## 1. Introduction

Gene therapy has received considerable interest since the 2006 Nobel prize in medicine [1]. In gene therapy, nucleic acids such as small interfering RNA (siRNA), messenger RNA (mRNA) or plasmid DNA (pDNA) are introduced into cells to degrade targeted genes or trigger the production of proteins such as antigens [2,3]. As such, gene therapy holds great promise for the treatment of cancers and vaccination against infectious diseases [4,5]. As a recent example, gene-based vaccines are the front runners in the race to become the first vaccine against the 2019 coronavirus (SARS-CoV-2) [6,7]. Despite the tremendous potential, translation of gene therapies to the clinic remains limited due to challenges associated with in vivo gene delivery [8]. Nucleic acids have molecular weights below the cut off of glomerular filtration by the kidney and are thus excreted rapidly when entering the systemic circulation [9]. Nucleic acids are also prone to degradation by enzymes and negatively charged, which limits their uptake in target tissues and cells [9]. As such, considerable efforts have been directed toward developing new gene delivery technologies to overcome these significant challenges [10].

Lipid nanoparticles (LNPs) are one of the most advanced technologies successfully developed to deliver nucleic acids [11,12]. LNPs encapsulating siRNA (Onpattro^®^) have been approved by the U.S. Food and Drug Administration (FDA) for use in humans [13]. LNP delivery technology is also widely used in the majority of nucleic acid vaccines currently under clinical trials [14]. The success of LNPs may inherit from their previous generation, that is liposomes. A variety of liposomal drugs have been successfully translated into clinics [15]. While liposomes have a simple bilayer structure and an empty water pocket in the core, LNPs possess a solid core comprising nucleic acids, cholesterols and lipids [16,17]. After a decade of research and development, LNP components have been optimized to achieve high delivery and transfection efficiency [18]. However, current LNP technology delivers nucleic acids to only a few tissues such as liver and muscle, limiting their potential applications and the effectiveness of nucleic acid vaccines or cancer immunotherapies [18,19].

To achieve highly effective nucleic acid vaccines or immunotherapies, it has been increasingly recognized that nucleic acids should be delivered to lymph nodes [20]. Lymph nodes have a large number of immune cells responsible for activating and regulating the adaptive immune response [21]. For instance, antigen-presenting cells such as CD103^+^ dendritic cells must traffic to draining lymph nodes to present tumour antigens to T cells, which then proliferate and migrate to the tumour to kill cancer cells [22]. CD8^+^ dendritic cells and CD169^+^ macrophages play an important role in initiating the antibody production process but are resident only in lymph nodes [23]. As such, delivering nucleic acids to lymph nodes allows presenting tumour or virus antigens to immune cells more effectively, leading to increasing antibody production efficiency and reducing side effects due to off-target transfections [24]. Such LNP delivery technology would pave the way for the development of safe and effective genetic vaccines.

Several strategies have been explored to improve targeted delivery to lymph nodes; however, none of the current strategies has proven both effective and practical [24]. For example, intranodal injection of LNPs into the lymph nodes is the direct route of administration but this strategy requires surgery or use of an ultrasound device, which is risky and impractical for community vaccination [22]. Furthermore, while nanoparticles can be guided by a magnetic field, functionalized with targeting agents or engineered with suitable size and charge, these strategies are complicated and not applicable for LNPs and in vivo gene delivery [21]. It is challenging to design LNPs for lymph node targeting because the surface of LNPs should have a negative charge and a large amount of poly(ethylene glycol) (PEG) chains to minimize interactions with negatively charged lymphatic vessels and interstitium, but on the other hand, should possess a positive charge and a low level of PEGylation to promote interactions with immune cells at the lymph nodes [25]. An innovative strategy to switch the surface property of LNPs when reaching the lymph node is urgently needed and highly beneficial.

PEGylation is a current gold standard in controlling the surface property of nanoparticles [26,27,28]. Although PEGylated drugs and nanoparticles may cause PEG immunogenicity and accelerated blood clearance (ABC) (see our review for more details) [26], there is still a long list of PEGylated drugs and nanoparticles approved for clinical treatments including Onpattro^®^, the first and only FDA-approved LNPs used for gene delivery [13]. In the Onpattro^®^ formulation, it has been reported that PEG chains can detach from the surface of LNPs during blood circulation, leading to targeting LNPs to the liver [29]. This strategy may minimize the PEG immunogenicity as no ABC phenomenon of the Onpattro^®^ has been reported. A similar strategy to target LNPs to lymph nodes and achieve highly specific transfection at these lymphatic organs, however, remains unattainable.

In this work, we hypothesized that the structure of PEG and its lipid tails might play an important role in switching the surface property of LNPs and promoting lymph node targeting and transfection. Tweens belong to a family of PEG surfactants with branched PEG chains attaching to lipid tails with different lengths, and therefore, are ideal candidates to test our hypothesis [30]. Tween 80 and Tween 20 have a similar branched PEG structure linking to an unsaturated C17 and a saturated C11 tail, respectively [31,32,33]. Importantly, both Tween 80 and 20 are biocompatible, have been clinically used in injectable formulations, can stabilize lipid bilayers in serum, and enhance gene delivery efficiency [34,35,36]. Therefore, we employed Tween 80 and 20 to replace a widely used PEG attaching to two stearoyl tails (PEG-DSPE) and subsequently studied the size, charge, stability and DNA encapsulation efficiency of the resulting LNPs. In addition, the in vivo transfection efficiency was also investigated to elucidate the effect of PEG and lipid structure on the selective organ targeting of LNPs. The results showed that Tween 80 and Tween 20 are not only good alternatives for PEG-DSPE but are also critical to the selective organ targeting capacity of LNPs.

## 2. Materials and Methods

### 2.1. Materials

Plasmid pNL1.1CMV (designed by Promega, Madison, WI, USA) was purchased from PlasmidFactory & Co. KG (Bielefeld, Germany). The ionizable aminolipid (6Z,9Z,28Z,31Z)-heptatriacont-6,9,28,31-tetraene-19-yl 4-(dimethylamino)-butanoate (DLin-MC3-DMA) was purchased from DC Chemicals. Helper lipid 1,2-distearoyl-sn-glycero-3-phosphocholine (DSPC), cholesterol, 2-distearoyl-sn-glycero-3-phosphoethanolamine-N-[methoxy(polyethylene glycol)-2000] (PEG-DSPE) and polyethylene glycol sorbitan monooleate (Tween 80) were obtained from Sigma-Aldrich. Poly(ethylene glycol) sorbitan monolaurate (Tween 20) was purchased from Croda International (Snaith, UK).

### 2.2. Methods

#### 2.2.1. Formulation of LNPs Containing pDNA

LNP-pDNA formulations were prepared using a previously described methodology [37]. Briefly, the lipid solution was prepared by dissolving the amino lipid (DLin-MC3-DMA), helper lipid (DSPC), cholesterol and PEG-DSPE/Tween 80/Tween 20 in absolute ethanol at a molar ratio of 52:8:37–38.5:3.0–1.5, respectively, and at a total concentration of 10 mM. The pDNA (pNL1.1CMV), expressing nanoluciferase (nLuc), concentration was determined using NanoDrop (Nd 3300 Fluorospectrometer Fluorescence, Thermo Scientific, Waltham, MA, USA) and was subsequently dissolved in 25 mM sodium acetate buffer (pH 4). The two solutions were mixed in a microfluidic chip using a staggered herringbone mixer (SHM)/Nanoassemblr (Precision Nanosystems, Vancouver, BC, Canada) at a fixed flow rate of 8 mL/min. All formulations were produced at 95.33 μg DNA per μmol lipid (corresponding to a N/P charge ratio of 6) unless otherwise stated. The LNPs were subsequently purified by dialyzing against 500× phosphate-buffered saline (PBS) at pH 7.4 using a Pur-A-Lyzer™ Maxi Dialysis Kit (0.1–3 mL, MWCO 6–8 kDa, Sigma-Aldrich, St. Louis, MI, U.S.) for at least 18 h. LNPs used for in vivo studies were further filtered (Millex 13 mm Durapore PVDF 0.45 μm, Merck, Fort Kenalworth, NJ, USA) and concentrated (Amicon Ultra-0.5 mL Centrifugal Filters 50 K, Merck) to a final pDNA concentration of 100 μg/mL and stored at 4 °C until use. All LNPs were stored at 4 °C until use.

#### 2.2.2. LNP Characterization

LNPs were characterized using dynamic light scattering (DLS) (Zetasizer Nano ZS, Malvern Instruments, Worcestershire, UK), as previously described [38]. DLS was used to determine the average particle size (average particle diameter in nm based on light scattering by intensity) with ZEN0040 disposable cuvettes (Malvern Panalytical) in PBS at a pH of 7.4. For zeta potential measurements, LNPs were diluted 10-fold with nuclease-free water and analysed using a DTS107 disposable folded capillary (Malvern Panalytical, Malvern, UK). Samples were analysed in triplicates and reported as the mean ± SD of the size by intensity measurements to account for multiple populations. One population was observed unless stated otherwise. DNA entrapment efficiency was quantified with a Quant-iT Pico-Green dsDNA assay kit (ThermoFisher Scientific, Waltham, Massachusetts). All LNP samples were diluted 10-fold in TE buffer and plated on a Corning^®^ 96 Well Solid Polystyrene Microplate (Sigma-Aldrich). The 1% Triton X-100 was added to the wells to lyse the LNP and release the entrapped pDNA. Control samples were not lysed and consisted of naked pDNA. The plate was incubated for 15 min at 40 °C, 300 rpm using a Thermomixer R (Eppendorf, Hamburg, Germany). Quant-iT PicoGreen reagent was added to all wells and the plate was further mixed at room temperature for 5 min. Fluorescence was measured on a EnVision 2103 Multilabel Reader (PerkinElmer precisely, Waltham, MA, USA) at excitation and emission wavelengths of 480 and 520 nm, respectively. Encapsulation efficiency was determined by measuring fluorescence upon addition of PicoGreen to LNPs and comparing it to the value obtained post-lysis; as shown below:

(1) $$\%\;\mathrm{EE}=\left(\frac{\mathrm{Lysed}\;\mathrm{LNP}-\mathrm{NonLysed}\;\mathrm{LNP}}{\mathrm{Lysed}\;\mathrm{LNP}}\right)x\;100$$

Each sample was analysed in 3 technical replicates. Results are stated as the mean ± SD. All formulations for in vivo use were tested for particle size, charge and DNA encapsulation were found to be between 85 and 185 m in size, with greater than 50% encapsulation.

#### 2.2.3. Analysis of Particle Stability

Fridge storage stability: LNPs were stored in Eppendorf tubes at 4 °C for up to three weeks. Particle size measurements were conducted for each sample at week 0 following dialysis and week 1–3. Samples were measured in triplicates. Data are reported as the mean ± SD.

Serum stability: LNPs encapsulated with pDNA were diluted 10-fold with PBS and was used as a positive control. Other LNPs were subjected to 10% of mouse serum. Both samples were incubated at 37 °C for 60 min, 300 rpm. Size and PDI were subsequently determined using DLS. Samples were measured in triplicates; reported data are represented as the mean ± SD.

#### 2.2.4. In vivo Gene Expression nLuc by LNPs

All experiments were approved by the Monash Institute of Pharmaceutical Sciences, Monash University, animal ethics committee and were conducted in accordance with the Australian and New Zealand Council for the Care of Animals in Research and Teaching guidelines (The approved code is 2020-23088-47465, 19 March 2020). Male BALB/c mice (age 6–9 weeks, weight range of 20–30 g) were maintained on a standard diet with free access to water were used in all experiments. Animals were housed on a 12 h light/dark cycle, at ambient temperatures (21–22 °C). In all experiments, we used *n* = 3 mice per group. All mice received a single dose of 5 µg of nLuc (50 μL) intramuscularly (IM) into the quadriceps, using 0.25 mm (31G) × 8 mm needle size (BD Ultra-Fine Insulin Syringes, Medshop Australia). Mice were anaesthetized using isoflurane (2–5%) at 95% oxygen and placed on a heated pad at 37 °C to maintain body temperature throughout anaesthesia. The level of isoflurane was introduced at a concentration range of 1–3% (97% oxygen) to maintain anaesthesia during LNP administration. Mice were sacrificed 24 h post-injection and organs were harvested by dissection and frozen (−80 °C) until further processing.

#### 2.2.5. Ex vivo Whole-Organ Bioluminescence Imaging

Bioluminescent imaging was performed on isolated organs using the In Vivo Imaging System Lumina (IVIS) Lumina II imaging system (PerkinElmer) to measure whole-organ luminescence, as previously described [39]. Briefly, dissected tissues were transferred to a 24-well plate and soaked in 40× Furimazine reagent (Nano-Glo^®^ Luciferase Assay Reagent, Promega Corporation) for 5 min at room temperature. All images were acquired using an exposure time of 1 min. Luminescence data were processed with Living Image software 4.3.1 and quantified in average radiance in units of photons per second per centimetre squared per steradian (p/s/cm^2^/sr) (PerkinElmer).

#### 2.2.6. NanoLuciferase (nLuc) Assay

Frozen organs were thawed and weighed before being placed in MACS M tubes (Mitenyi Biotech Australia Pty Ltd., NSW, Australia). A volume of 1 mL of Glolysis buffer (Nano-Glo^®^ Luciferase Assay, Promega Corporation) was added to the organs before samples were homogenized using a GentleMACS Octo Dissociator (Mitenyi Biotech Australia Pty Ltd., NSW, Australia) running a standard program (program Protein 1, M-tube mode). Hereafter, tissue homogenates were centrifuged for 5 min at 3000× *g*, 4 °C to remove tissue debris. Approximately 800 mL of supernatant was collected and further centrifuged for 5 min, 10,000× *g* at 4 °C. The supernatant was used to weight normalize the tissue according to the average weight of two lymph nodes. A volume of 100 μL of each normalized tissue lysate was plated on OptiPlate 96-well plate (ProxiPlate-96, White Opaque 96-shallow well Microplate, Perkin Elmer). A volume of 100 μL of substrate Nano-Glo substrate (Nano-Glo^®^ Luciferase Assay System, Promega) was added to these well using a multichannel pipette. The plate was mixed at ambient temperature, 300 rpm for 15 min. NLuc expression in tissues was analysed with EnVision 2103 Multilabel Reader (PerkinElmer) at wavelength 460 nm. All the relative luminescence units (RLU) were converted and normalized to the amount of nLuc enzyme per mg of the tissue. Reported data are presented as the mean ± SEM.

#### 2.2.7. Statistical Analysis

Statistically significant differences were determined by ANOVA followed by Dunnett’s test and Turkey’s test for multiple comparisons, using a significance level of α = 0.05. Statistical calculations were performed using GraphPad Prism software, version 8.2 for macOS (GraphPad Software Inc.,San Diego, CA, USA).

## 3. Results and Discussion

### 3.1. Synthesis of LNPs Encapsulating Plasmid DNA

**Figure 1:**
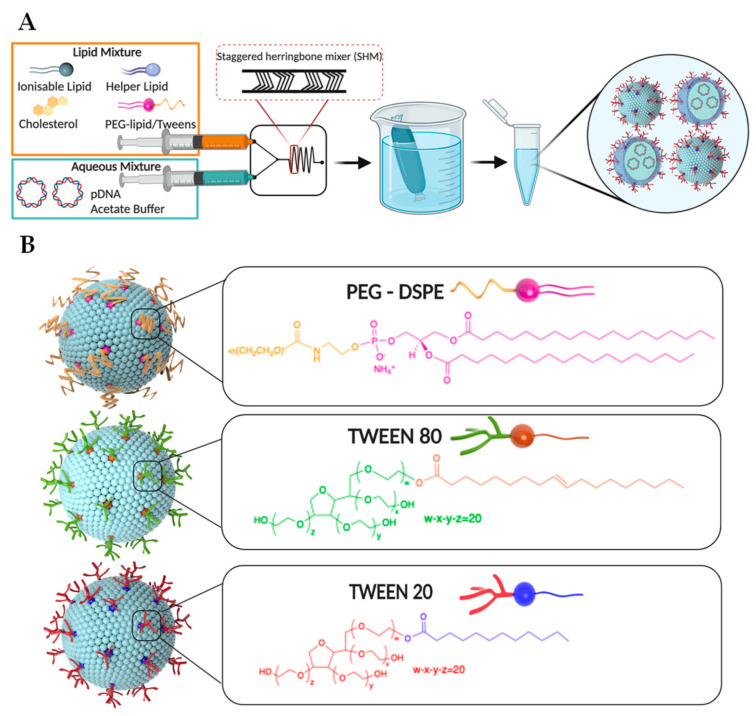
Schematic presentation of (**A**) a scalable, rapid, and reproducible microfluidic mixing technology (NanoAssemblr^®^) used in this work to prepare LNPs and (**B**) three types of LNPs stabilized by PEG-DSPE, Tween 80, or Tween 20.

In this work, LNPs encapsulating pDNA were prepared using a scalable, rapid, and reproducible microfluidic mixing technology (NanoAssemblr^®^) [40]. Key to this technology is a specially designed microfluidic chip with two inlets and a staggered herringbone micromixer (SHM) area (Figure 1A) [41]. All LNP components including an ionizable lipid (Dlin-MC3-DMA), a helper lipid (DSPC), cholesterol, and PEG-DSPE (or Tweens) were dissolved in ethanol and injected in one inlet. pDNA in acetate buffer was simultaneously injected to another inlet. The two solutions underwent rapid, chaotic mixing at the SHM interface inside the microfluidic chip where the amine head groups of the ionizable lipid were protonated, allowing complexation with the negatively charged pDNA. Other components become insoluble in the ethanol/acetate buffer mixture and self-assemble to form initial LNPs with pDNA encapsulated. The final LNP products were obtained by dialyzing the solutions collected from the microfluidic chip against PBS buffer overnight (18 h). This simple, two-step procedure facilitates scalable, rapid production of LNPs with minimal risk of contamination. It should be noted that Inflexal^®^ V, an approved liposomal vaccine, was discontinued in 2014 due to bacterial contamination during a complicated and multistep manufacturing process [42]. Consequently, simplified and scalable production of LNPs is particularly important for their potential applications in vaccines and immunotherapies [43].

Except for PEG and pDNA, all other components of LNPs (ionizable lipid, cholesterol and helper lipid) were selected based on the FDA-approved formulation, Onpattro^®^ [13]. DLin-MC3-DMA is an ionizable lipid utilized for complexation with pDNA and is the main component determining LNP structure [44]. As DLin-MC3-DMA is the main component of the FDA-approved formulation, this ionizable cationic lipid is safe for use in humans, and therefore selected for use in this work. Cholesterol enhances LNP stability and promotes endosomal escape [45]. Helper lipid DSPC, as the name suggests, aids in the formation of LNP structure [46]. The molar ratio of DLin-MC3-DMA:cholesterol:DSPC was kept at 52:38.5:8, found to be optimum for achieving high in vivo transfection efficiency [47]. In this work, pDNA encoding for nanoluciferase (NLuc) was selected to probe the transfection efficiency of LNPs due to its bright luminescence and low background noise. The ratio of nitrogen to phosphate (N/P) used in this work was 6, as a lower N/P ratio resulted in decreased encapsulation efficiency and a high N/P ratio reduced tolerance (data not shown). To elucidate the role of PEG and its lipid structure on lymph node targeting and transfection, we chose Tween 80 and Tween 20 to replace the standard PEG-DSPE component and synthesized three types of LNPs (Figure 1B). The molar ratio of PEG-DSPE, Tween 80, or Tween 20 in initial LNP formulations was 1.5% (as typically used in the Onpattro^®^ and other LNPs), which was then doubled to further elucidate the potential effects of the PEG component on LNP biophysical properties, tissue localization and transfection efficiency. All synthesized LNPs were carefully characterized to evaluate their size, surface charge, pDNA encapsulation efficiency, and stability before being applied to in vivo transfection studies.

### 3.2. Characterization of LNPs

**Figure 2:**
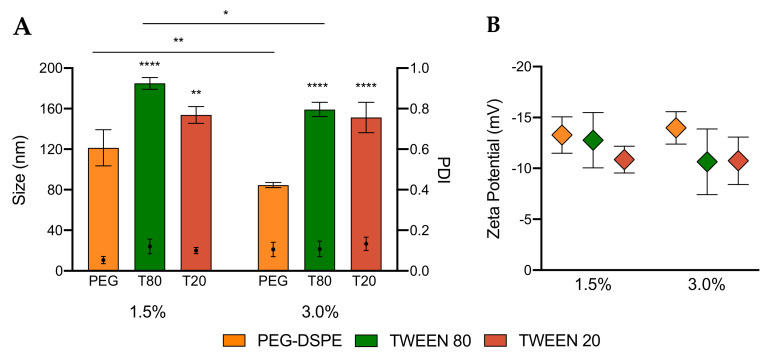
(**A**) Hydrodynamic diameter (size), polydispersity (PDI) and (**B**) zeta potential of LNPs made with 1.5% and 3% of PEG-DSPE, Tween 80 and Tween 20. Two-way ANOVA was performed on graph A. * *p* < 0.05; ** *p* < 0.01; **** *p* < 0.0001.

To elucidate the effect of PEGylation by Tweens and PEG-DSPE, we first characterized the size, polydispersity index (PDI) and surface charge of LNPs by dynamic light scattering (DLS) and zeta potential measurements. The data reported in Figure 2 are average values of nine measurements per sample (three measurements of three batches), allowing us to evaluate not only the range of size and charge of each sample but also the reproducibility of the synthesis method. The data in Figure 2A and Table S1 (in Supplementary Materials) demonstrate that both Tween 80 and Tween 20 can reproducibly promote the formation of LNPs with Z-average hydrodynamic diameters ranging from 150 to 200 nm. Interestingly, these results also show that even the short lipid tail (11 carbons) of Tween 20 is sufficiently hydrophobic to anchor onto the surface of LNPs and its short PEG chain (20 units of ethylene glycol) can sterically stabilize these particles (Figure 1B). Tween LNPs (both Tween 80 and Tween 20) exhibit larger hydrodynamic sizes comparative to LNPs stabilized by PEG-DSPE of approximately 120 and 80 nm for 1.5% and 3%, respectively. This difference may be related to both the length of PEG and its hydrophobic tails. Tweens have 20 units of ethylene glycol and a single lipid tail, while PEG-DSPE is made of 45 units and two alkyl tails. The longer PEG chains and lipid tails were found to facilitate better stabilization and a more compact structure. Increasing the molar ratio of Tweens from 1.5% to 3% did not significantly decrease the particle size, suggesting that the two long lipid tails of PEG-DSPE play a key role in controlling the size of LNPs formulated with PEG lipids. Even though PEG-DSPE has a longer PEG chain and lipid tails, PDI values of all LNPs formed were not significantly different when compared to Tween LNPs. Low PDI values (below 0.2) reveal that Tween 80 and Tween 20, despite having shorter PEG chains, can prevent LNPs from aggregating thereby promoting the production of uniform LNPs analogous to the gold standard PEG-DSPE LNPs. These low PDI values also suggest that the short, branched PEG chains of Tweens may cover a similar surface area and sterically stabilize LNPs as well as the long, linear PEG chains. All synthesized LNPs were also shown to have relatively similar zeta potential values, ranging from −7 to −17 mV (Figure 2B), which is typical for PEGylated nanoparticles [48]. In summary, the characterization of LNPs by DLS confirms that Tween 80 and Tween 20 are viable alternatives to PEG-DSPE in the production of stable LNPs.

### 3.3. Encapsulation Efficiency and Stability of LNPs

**Figure 3:**
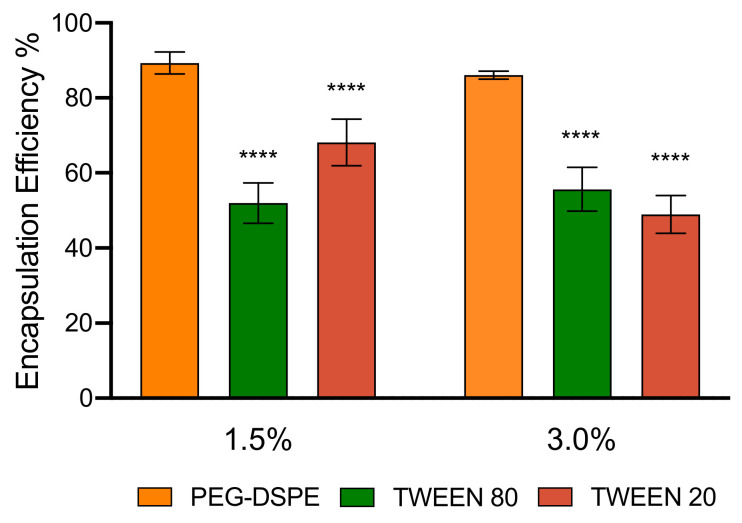
pDNA encapsulation efficiency of LNPs made with 1.5% and 3% of PEG-DSPE, Tween 80 and Tween 20. One-way ANOVA was performed followed by Dunnett’s test for multiple comparisons. **** *p* < 0.0001.

After confirming the formation and stabilization of LNPs by using Tweens 80 and Tween 20, we further interrogated the capacity for LNPs PEGylated by Tweens to encapsulate a pDNA cargo. PicoGreen^®^ dye, which binds strongly and specifically to free double-stranded DNA, was used to quantify the encapsulation efficiency of pDNA [49]. It should be noted that PicoGreen^®^ is a hydrophilic dye and, in principle, cannot diffuse through the LNP core. As such, when PicoGreen^®^ is added to LNP solutions, only non-encapsulated pDNA can bind to the dye, allowing the quantification of the free pDNA outside of LNPs. To measure the total amount of pDNA both inside and outside of LNPs, Triton X-100 was added and the mixture was heated at 40 °C for ten minutes to break the LNPs and release the encapsulated DNA prior to adding the PicoGreen^®^ dye. By quantifying the total amount of pDNA and free pDNA outside of LNPs, the encapsulation efficiency or the percentage of encapsulated pDNA was calculated using Equation (1), and the data are presented in Figure 3 and Table S1. LNPs with 1.5% and 3% of PEG-DSPE had encapsulation efficiency of approximately 80% to 90%. Interestingly, LNPs PEGylated by Tween 80 and Tween 20 encapsulated relatively similar amounts of pDNA. However, both Tween LNPs had a lower encapsulation efficiency than those made with PEG-DSPE, indicating that the branched structure and shorter length of PEG chains may affect the encapsulation efficiency. This result presents an interesting new angle to theorized mechanics of LNP encapsulation, in which only the ionizable lipid is responsible for complexing with pDNA and determining the encapsulation efficiency, and the PEG component mainly provides stability and control surface property of the LNPs. We speculate that the branched and short PEG chains of Tweens may render the LNPs less compact (as also indicated via larger LNP size) and therefore allow some weakly bound pDNA molecules to diffuse out of the LNPs during dialysis. In addition, the lower encapsulation efficiency of Tween LNPs may be also related to a proportion of pDNA binding on the surface of the LNPs or the dye may be able to penetrate to the loosen structure of the LNPs. In short, this work demonstrates that LNPs produced from Tweens are capable of encapsulating pDNA and forming LNPs with sizes and charges suitable for in vivo gene delivery.

**Figure 4:**
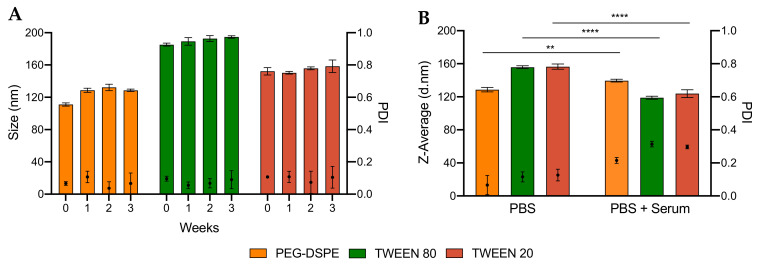
Stability of LNPs PEGylated with 1.5% PEG-DSPE, Tween 80 and Tween 20 (**A**) over 3 weeks and (**B**) in mouse serum. One-way ANOVA was performed on graph B, followed by Turkey’s test for multiple comparisons. ** *p* < 0.01; **** *p* < 0.0001.

We next compared the stability of LNPs made from DSPE-PEG and Tweens. We evaluated two types of stability: during long-term storage and in the presence of mouse serum (Tables S2 and S3). These stability tests are particularly important as the transfection efficiency of LNPs could be significantly reduced if LNPs aggregate during storage or after administration. Tweens have shorter PEG chains and lipid tails than PEG-DSPE and therefore LNPs stabilized by Tweens could theoretically be less stable. Surprisingly, we found that the size and PDI of all LNPs did not significantly change even after storing for up to 3 weeks at 4 °C (Figure 4A and Figure S1). These data indicate that, despite having shorter PEG chain and lipid tails, Tween 80 and Tween 20 can stabilize LNPs as well as PEG-DSPE in 3 weeks. Furthermore, Figure 4B and Table S4 show that all LNPs synthesized were stable in serum. Unlike long-term storage, the PDI of LNPs in serum increased to over 0.2 but remained below 0.3; and the average particle size of LNPs in serum decreased when compared to LNPs stored in PBS. These changes were also observed for PEG-DSPE, a gold standard known for well-stabilizing LNPs, which suggests that small proteins present in serum affected the DLS measurements leading to the smaller particles size and the higher PDI. In addition, no DNA leakage from LNPs during storage for 3 weeks was found, suggesting that both Tweens and PEG-DSPE are good stabilizers for pDNA LNPs (Figure S2). Taken together, we found that both Tweens 80 and 20 could stabilize LNPs for at least 3 weeks in PBS buffer and even in a complex biological milieu, which is encouraging for in vivo gene delivery applications.

### 3.4. In Vivo Transfection of LNPs Stabilized by PEG-DSPE

After the synthesis and characterization of LNPs, we studied in vivo transfection of LNPs encapsulating pDNA in mice. LNP containing pDNA was intramuscularly injected into the quadricep muscle of BALB/c mice. The intramuscular injection route is selected in this work as it is widely used for administration of vaccines in the clinic and is one of the most suitable routes allowing LNPs access to the lymph nodes via lymphatic vessels. A total of 24 h after injection, the following 8 tissues were harvested, homogenized, and analysed for NLuc gene expression: right draining lymph node (D-LN), left non-draining lymph node (L-LN), right muscle (RM), liver (LV), spleen (SP), kidneys (KD), heart (HT) and lung (LG). To quantify the level of transfection (the amount of NLuc protein produced in tissues sampled), Nano-Glo^®^ Luciferase substrate and buffer were added and the luminescence relative light unit (RLU) was recorded using a plate reader. The RLU signal is linearly correlated with the amount of NLuc protein produced from pDNA expression. As such, the high RLU signal indicates high DNA transfection efficiency. The RLU was normalized to the same mass of tissue (RLU/mg of tissue, Figure S3 and Table S4), allowing the direct comparison of transfection efficiency in different organs without confounding effect of variation in tissue mass (for example, without normalization, the liver is heavier than the lymph nodes, so it may show higher RLU signal even with a lower transfection efficiency). Logarithmic-scale and non-normalized data are also shown in Figures S4 and S5. To visually observe the luminescence, extracted tissues were incubated with Furimazine reagent and then imaged by the IVIS^®^ Spectrum imaging system.

**Figure 5:**
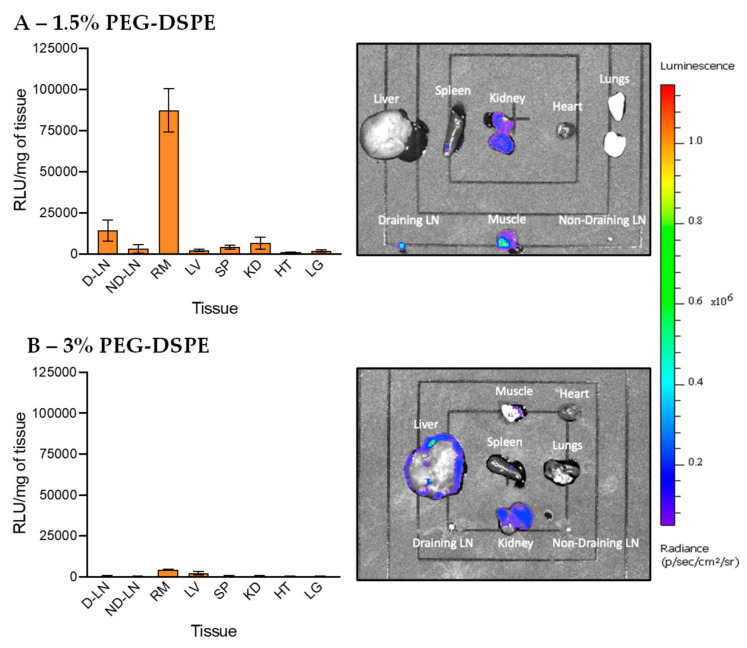
In vivo transfection of LNPs stabilized by PEG-DSPE at (**A**) 1.5% and (**B**) 3%. The graphs on the left show the luminescence relative light unit (RLU) of homogenized tissues recorded by the plate reader and the right IVIS images show the luminescence of whole tissues. The abbreviations of tissues are draining lymph node (D-LN), non-draining lymph node (ND-LN), right muscle (RM), liver (LV), spleen (SP), kidney (KD), heart (HT) and lungs (LG).

Data in Figure 5 show the in vivo transfection efficiency of LNPs stabilized by PEG-DSPE (1.5% and 3%). LNPs with 1.5% of PEG displayed the highest amount of gene expression in the muscle, as shown by strong luminescence intensity in IVIS imaging and very high RLU signal detected by the plate reader. To a much lower extent, transfections had occurred in the draining lymph node and kidneys. Little to no luminescence signal was found in the other tissues. These data show that this standard LNP formulation has high transfection at the injection site (muscle) and only a very small proportion of LNPs were able to migrate and transfect cells in the draining lymph node and kidneys. IVIS imaging in Figure 5A also shows that PEG-DSPE LNPs did not transfect evenly in the muscle, confirming that a large proportion of PEG-DSPE LNPs was not able to migrate away from the injection site. Interestingly, we found that when increasing the amount of PEG-DSPE to 3%, the transfection efficiency was noticeably reduced, and almost no transfection was found in all the organs harvested (Figure 5B). As all other components of LNPs remained unchanged and the pDNA encapsulation efficiency was relatively similar, this finding highlights the strong effect of PEGylation on the transfection efficiency of LNPs. An excessive amount of PEG chains on the surface of LNPs may prevent their internalization into cells or inhibit endosomal escape, leading to low transfection efficiency. Our finding is also concordant with previous work showing that 1.5% of PEG surfactant is optimal for achieving high in vivo transfection efficiency [50]. In short, the data show that using 1.5% PEG-DSPE to PEGylate LNPs leads to high transfection at the injection site post-intramuscular administration and little gene expression in the lymph nodes and kidneys, suggesting that PEG-DSPE is not a suitable component of pDNA LNPs used for vaccines and immunotherapies in which lymph node targeting is highly beneficial.

### 3.5. In Vivo Transfection of LNPs Stabilized by Tween 80 and Tween 20

**Figure 6:**
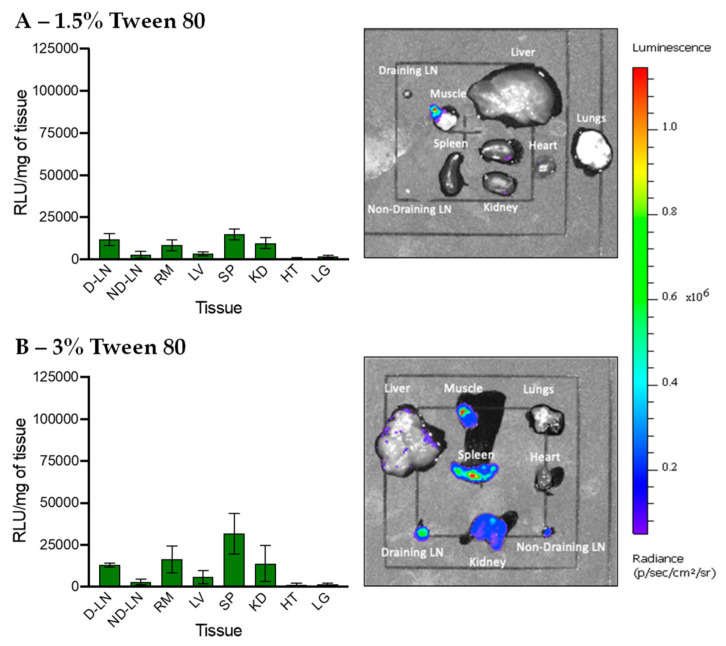
In vivo transfection of LNPs stabilized by Tween 80 at (**A**) 1.5% and (**B**) 3%. The graphs on the left show the luminescence relative light unit (RLU) of homogenized tissues recorded by the plate reader and the right IVIS images show the luminescence of whole tissues. The abbreviations of tissues are draining lymph node (D-LN), non-draining lymph node (ND-LN), right muscle (RM), liver (LV), spleen (SP), kidney (KD), heart (HT) and lungs (LG).

LNPs PEGylated by Tween 80 were next injected intramuscularly to study their transfection efficiency. LNPs stabilized by 1.5% Tween 80 have a relatively low transfection efficiency compared to LNPs PEGylated with 1.5% PEG-DSPE, especially in the muscle (Figure 6A and Figure S6A). This result suggests that not only the amount of PEG chains but also the PEG structure plays a critical in in vivo transfection efficiency of LNPs. With the same number of polymer chains, branched PEG may be able to cover a larger surface area of LNPs than linear PEG, leading to reduced cellular interactions and/or endosomal escape and as a result, lower transfection efficiency. Interestingly, Figure 6B and Figure S6B show that increasing the Tween 80 to 3% results in higher transfection in the spleen, although the overall transfection efficiency remains relatively low. LNPs PEGylated with 3% Tween 80 had a smaller size and lower transfection at the injection site than those with 1.5% and thus, may be able to traffic from the injection site to the spleen more. Targeted delivery of nucleic acids to the spleen is important in developing more effective immunotherapies, but is often achieved via the intravenous injection route. Therefore, LNPs PEGylated with 3% Tweens 80 reported in this work may pave the way for developing new immunotherapies targeting the spleen via intramuscular administration, which is preferred over the intravenous injection route because it enables at-home injection, improves quality of life, and reduces health care costs [51]. 

**Figure 7:**
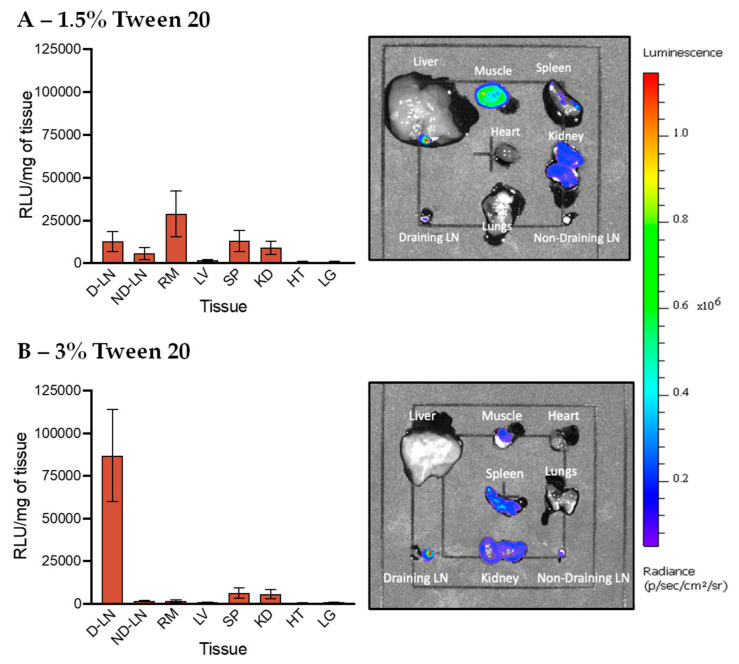
In vivo transfection of LNPs stabilized by Tween 20 at (**A**) 1.5% and (**B**) 3%. The graphs on the left show the luminescence relative light unit (RLU) of homogenized tissues recorded by the plate reader and the right IVIS images show the luminescence of whole tissues. The abbreviations of tissues are draining lymph node (D-LN), non-draining lymph node (ND-LN), right muscle (RM), liver (LV), spleen (SP), kidney (KD), heart (HT) and lungs (LG).

Finally, Tween 20 was used to PEGylate LNPs for in vivo pDNA delivery, and the transfection efficiency results are shown in Figure 7. We found that LNPs with 1.5% Tween 20 exhibited reduced transfection efficiency when compared to 1.5% PEG-DSPE LNPs, but their gene expression in the muscle was higher than LNPs stabilized by 3% PEG-DSPE and Tween 80. Importantly, we discovered that 3% Tween 20 LNPs exhibited very high gene expression in the draining lymph nodes while little to no transfection was found in the other tissues. This result suggests that the structure of lipid tails play a critical role in selective organ targeting after intramuscular injection. Similar to Tween 20, lipid tails of PEG-DSPE are also saturated; but PEG-DSPE LNPs are mainly transfected in the muscle. Therefore, we speculate that the length of the lipid tail but not its saturated nature is more likely to contribute to the high transfection efficiency in the lymph nodes, although in such a complex LNP system, the effect of PEG branching or the length and the degree of lipid unsaturation may all play a role collectively. The short lipid tail (11 carbons) may allow Tween 20 chains to detach from the LNP surface after trafficking to the lymph nodes, while Tween 80 with a long tail (17 carbons) and PEG-DSPE with two long tails (2 × 17 carbons) might remain on the LNP surface, and hence, did not exhibit high transfection at the lymph node. In addition, PEGylating LNPs with more Tween 20 chains on the surface (3%) could limit interactions of LNPs with cells at the injection site, thereby allowing more LNPs to traffic to the lymph node. Comparatively, 1.5% Tween 20 LNPs might have fewer Tween chains remained attached on the LNP surface after injection, resulting in higher transfection in the muscle but lower gene expression in tissues that are far from the injection site. Our finding is in good agreement with previous work showing that short lipid tails could be removed from LNP surface during blood circulation, leading to liver targeting [29]. However, lymphatic fluids have a much slower flow rate comparative to blood, so the impact on the de-absorption of PEG-lipid may differ [52,53,54]. A microfluidic platform mimicking the lymphatic system will be used in future studies to study this effect [55,56]. In this work, we demonstrate that the structure of PEG chains and lipid tails is crucial in selective organ targeting after intramuscular administration. As Tween 20 is biocompatible, low cost and readily available, PEGylation of LNPs by Tween 20 represents a much simpler and more practical method for improving lymph node targeting when comparative to intranodal injection or other previously developed techniques.

## 4. Conclusions

We report an innovative PEGylation method using Tween 20 to improve targeted gene delivery of LNPs to the lymph nodes after intramuscular administration. Tween 20 with a short lipid tail and a branched PEG chain was used to successfully stabilize LNPs encapsulating pDNA, and the resulting LNPs with 3% of Tweens 20 exhibited highly specific transfection in the lymph nodes. We also found that 1.5% Tween 20 LNPs had much lower tissue-specific gene expression. In addition, Tween 80 with a longer lipid tail could be used to form stable LNPs for spleen targeting but with a relatively low transfection efficiency. PEG-DSPE, a gold standard in LNP formulation consisting of a linear PEG chain and two lipid tails, facilitated the formation of LNPs. The resulting PEG-DSPE LNPs at 1.5% were shown to be transfected mainly at the injection site, while little to no transfection was observed when using this formulation at 3%. These findings confirm that PEGylation is critical in achieving selective organ targeting and the high in vivo transfection efficiency of LNPs, even though the ratio of PEG component is the lowest in LNP formulations. Taken together, our work may advance the field of targeted gene delivery for potential applications in vaccines and immunotherapies by providing a new, simple and practical PEGylation technology for the rapid and scalable production of LNPs for lymph node targeting.

## Supplementary Materials

The following are available online at https://www.mdpi.com/1999-4923/12/11/1068/s1, Table S1: Particle characterization data and pDNA encapsulation efficiency; Tables S2 and S3: Stability data; Figures S1 and S2: Stability data; Table S4: In vivo transfection data; Figures S3, S4, S5 and S6: In vivo transfection data.
