# Supplementary material for: The Importance of Poly(ethylene glycol) and Lipid Structure in Targeted Gene Delivery to Lymph Nodes by Lipid Nanoparticles
Source: Pharmaceutics. 2020 Nov 9;12(11):1068. doi: 10.3390/pharmaceutics12111068 (PMC7695259; doi:10.3390/pharmaceutics12111068)
Supplement: Supplementary file 1 [file pharmaceutics-12-01068-s001.pdf]

pharmaceutics

Supplementary Materials: The Importance of Poly(ethylene glycol) and Lipid Structure in Targeted Gene Delivery to Lymph Nodes by Lipid Nanoparticles

Danijela Zukancic, Estelle J. A. Suys, Emily H. Pilkington, Azizah Algarni, Hareth Al-Wassiti and Nghia P. Truong

Table S1. Particle Characterisation data.

	Size (nm)	PDI	Z-Potential (mV)	EE %	Size (nm)	PDI	Z-Potential (mV)	EE %
	1.5 PEG – DSPE LNP				3.0 PEG – DSPE LNP			
1	128.50	0.05	−12.60	92.00	87.89	0.09	−11.80	86.00
	124.60	0.04	−14.90	93.00	85.42	0.09	−14.50	88.00
	99.51	0.06	−15.60	93.00	85.08	0.07	−13.90	86.00
2	99.14	0.08	−12.40	86.00	88.20	0.08	−12.00	87.00
	97.89	0.07	−13.80	90.00	84.76	0.10	−14.10	85.00
	128.00	0.06	−15.20	86.00	84.80	0.07	−16.50	85.00
3	137.00	0.03	−10.00	88.00	83.00	0.13	−16.00	85.00
	143.20	0.04	−12.00	86.00	81.59	0.16	−14.00	87.00
	134.60	0.05	−13.00	90.00	81.00	0.16	−13.00	86.00
	1.5 Tween 80				3.0 Tween 80			
1	191.90	0.11	−14.20	57.00	152.20	0.07	−7.50	55.00
	191.60	0.12	−15.70	46.00	150.20	0.09	−7.60	49.00
	189.20	0.08	−17.20	58.00	148.10	0.07	−7.70	50.00
2	183.50	0.09	−10.60	43.00	167.80	0.06	−9.00	49.00
	185.20	0.11	−10.80	49.00	163.80	0.14	−11.00	55.00
	187.26	0.09	−8.90	55.00	160.30	0.10	−9.00	55.00
3	183.20	0.17	−10.90	49.00	165.40	0.15	−15.00	63.00
	177.10	0.16	−12.50	57.00	162.30	0.14	−15.00	63.00
	175.70	0.17	−14.10	54.00	162.20	0.16	−14.00	62.00
	1.5 Tween 20				3.0 Tween 20			
1	156.50	0.10	−11.40	73.00	151.40	0.13	−7.68	42.00
	152.80	0.11	−11.80	73.00	146.70	0.12	−11.20	42.00
	147.40	0.11	−11.80	76.00	145.60	0.13	−11.10	43.00
2	166.60	0.11	−8.00	71.00	140.90	0.18	−13.70	53.00
	163.00	0.12	−10.00	60.00	136.10	0.17	−13.00	52.00
	160.40	0.07	−12.00	73.00	132.00	0.16	−12.00	52.00
3	147.90	0.10	−11.00	62.00	170.80	0.14	−9.00	52.00
	146.10	0.10	−11.70	62.00	168.80	0.09	−7.00	52.00
	143.50	0.10	−10.00	63.00	169.90	0.08	−12.00	53.00

Table S2. Stability during storage.

1.5% PEG-DSPE		3.0% PEG-DSPE		1.5% T80		3.0% T80		1.5% T20		3.0% T20	
Size (nm)	PDI	Size (nm)	PDI	Size (nm)	PDI	Size (nm)	PDI	Size (nm)	PDI	Size (nm)	PDI
Week 0											
113.30	0.05	88.20	0.08	183.50	0.09	165.40	0.15	156.50	0.10	151.40	0.13
110.20	0.07	84.76	0.10	185.20	0.11	162.30	0.14	152.80	0.11	146.70	0.12
109.70	0.07	84.80	0.07	187.26	0.09	162.20	0.16	147.40	0.11	145.60	0.13
Week 1											
127.60	0.08	95.06	0.09	188.20	0.08	131.70	0.15	152.20	0.13	152.60	0.17
126.70	0.10	94.45	0.06	194.30	0.04	128.10	0.10	150.00	0.07	146.90	0.11
131.70	0.15	92.25	0.00	185.20	0.05	129.00	0.08	149.00	0.12	148.40	0.10
Week 2											

136.10	0.02	93.54	0.11	196.00	0.10	126.60	0.15	154.20	0.15	152.00	0.13
132.70	0.01	94.16	0.12	193.40	0.05	127.70	0.14	157.90	0.01	152.50	0.16
128.10	0.08	93.22	0.07	188.70	0.05	128.00	0.04	155.60	0.06	148.80	0.13
Week 3											
130.30	0.06	92.08	0.17	195.00	0.15	123.40	0.11	163.40	0.03	151.40	0.06
127.60	0.14	92.28	0.07	193.20	0.05	120.50	0.17	162.50	0.11	145.60	0.09
128.00	0.01	90.73	0.07	196.10	0.06	119.00	0.16	149.40	0.17	146.90	0.11

Table S3. Serum stability.

1.5% PEG-DSPE		3.0% PEG-DSPE		1.5% Tween 80		3.0% Tween 80		1.5% Tween 20		3.0% Tween 20	
Size (nm)	PDI	Size (nm)	PDI	Size (nm)	PDI	Size (nm)	PDI	Size (nm)	PDI	Size (nm)	PDI
PBS											
127.6	0.129	121.9	0.03	157.2	0.089	183.5	0.071	158.9	0.144	194	0.132
126.7	0.053	117.4	0.111	154	0.109	173.8	0.098	152.9	0.15	186.7	0.032
131.7	0.015	120.3	0.022	156.2	0.148	173.9	0.128	157.5	0.086	184	0.159
Serum											
141	0.202	95.48	0.259	118.6	0.332	148.9	0.238	129	0.292	161.2	0.277
139.9	0.208	93.84	0.279	120.9	0.301	147.8	0.194	119.7	0.288	168.4	0.233
137.8	0.235	94.76	0.265	117.3	0.308	147	0.261	122.7	0.309	159.1	0.272

Table S4. Plate Reader In-vivo data represented as RLU/mg tissue.

Tissue	1.5% PEG-DSPE			3.0% PEG-DSPE		
D-LN	17602.90	2222.20	23666.70	727.30	208.70	80.80
ND-LN	800.00	8631.60	666.70	363.60	208.70	80.80
RM	80858.70	112991.50	68333.30	4242.40	4660.90	3692.30
LV	1431.10	2222.20	3666.70	4000.00	695.70	1679.00
SP	2432.90	3760.70	6666.70	1212.10	139.10	80.80
KD	13738.80	1709.40	5000.00	727.30	208.70	565.70
HT	1001.80	1025.60	1333.30	484.80	69.60	80.80
	1.5% Tween 80			3.0% Tween 80		
D-LN	17066.70	13377.80	4837.20	10917.60	14792.50	13186.80
ND-LN	1292.90	6545.50	434.10	738.50	6339.60	1582.40
RM	12307.70	11151.50	2170.50	22754.90	26264.20	703.30
LV	4007.20	5090.90	1054.30	2289.80	13584.90	1582.40
SP	17173.50	19151.50	8682.20	13023.30	54037.70	28044.00
KD	6583.20	16000.00	6387.60	3005.40	35320.80	3428.60
HT	715.60	1454.50	310.10	715.60	3018.90	87.90
	1.5% Tween 20			3.0% Tween 20		
D-LN	23619.00	3428.60	11105.90	137481.50	44952.40	78285.70
ND-LN	3428.60	12800.00	847.10	2469.10	1238.10	1333.30
RM	51809.50	29028.60	5364.70	1185.20	2952.40	285.70
LV	2285.70	2285.70	658.80	691.40	952.40	666.70
SP	4571.40	25371.40	9129.40	3851.90	12285.70	2666.70
KD	13714.30	11885.70	1317.60	1975.30	10571.40	3904.80
HT	1523.80	457.10	94.10	493.80	666.70	285.70

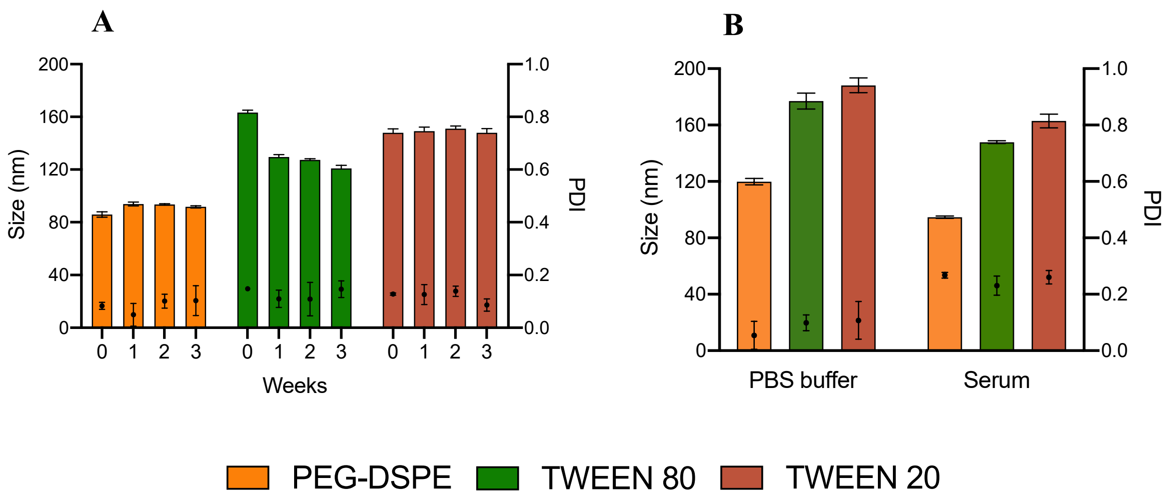

Figure S1. Stability. (**A**) Fridge stability for 1.5% LNP. (**B**) Serum stability for 1.5% LNP.

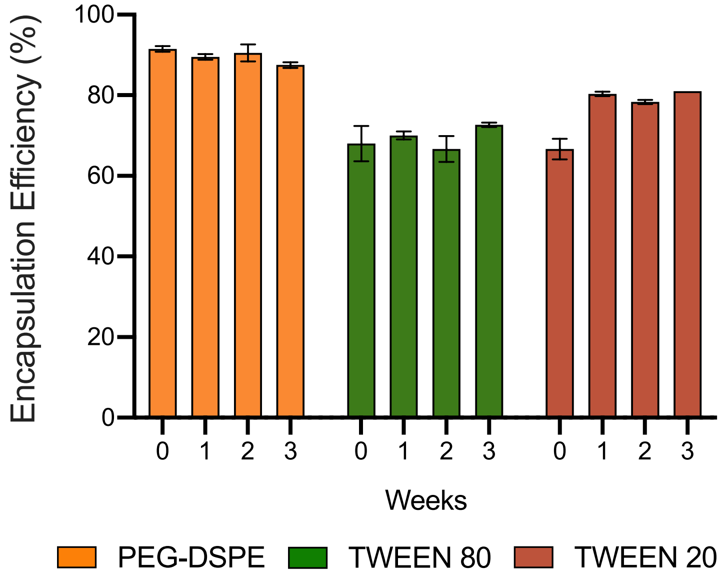

Figure S2. Stability (DNA leakage).

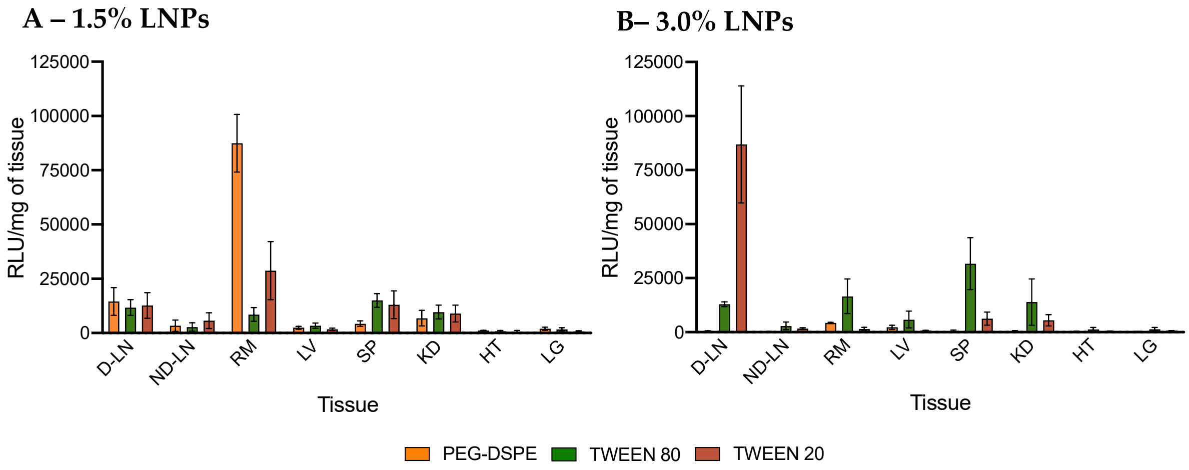

Figure S3. Plate Reader In-vivo data represented as (RLU/mg tissue) for all samples: (**A**) 1.5% and (**B**) 3%

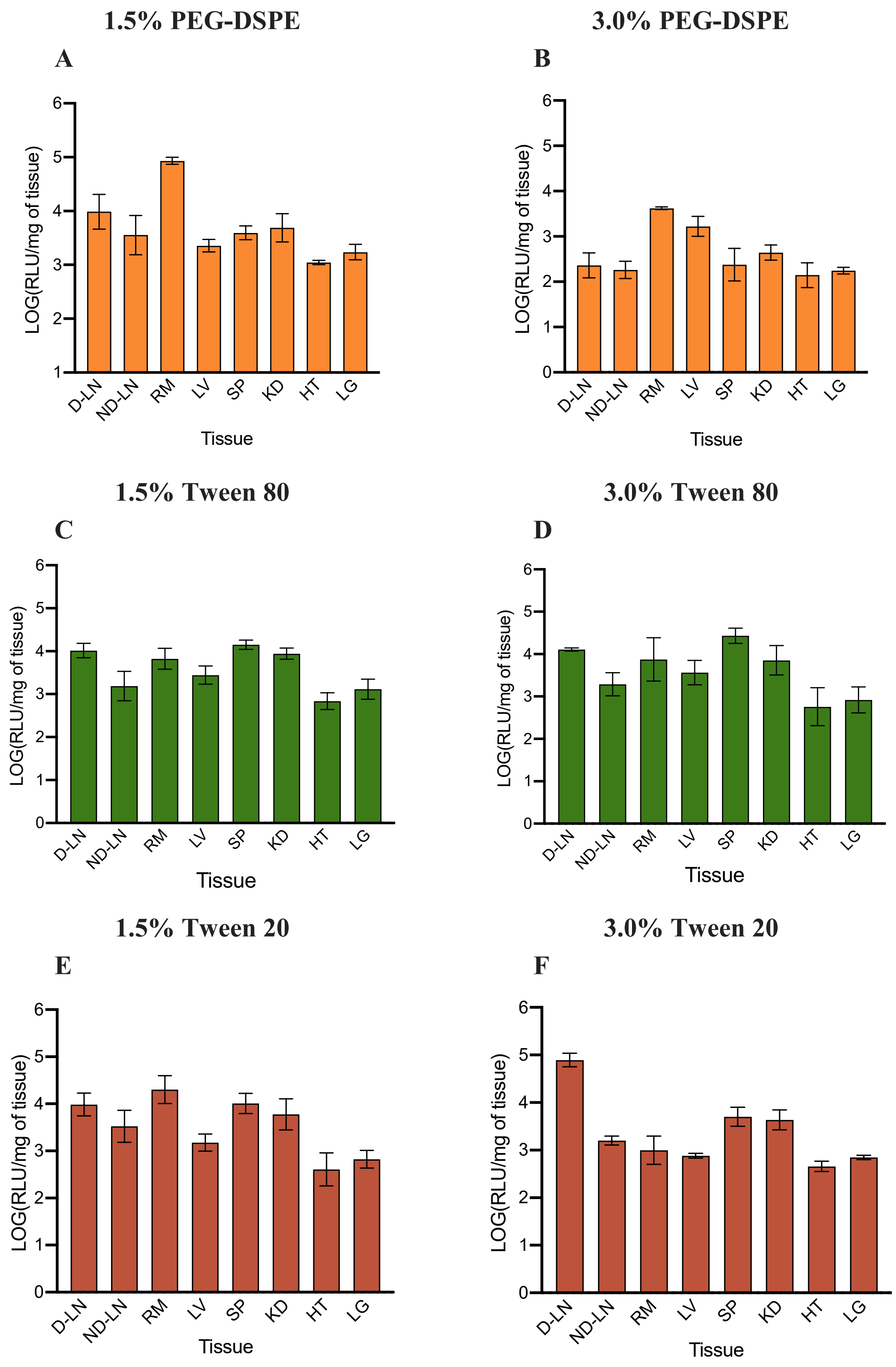

Figure 4S. Plate Reader In-vivo data represented as log(RLU/mg tissue): (**A**) 1.5% PEG-DSPE, (**B**) 3% PEG-DSPE, (**C**) 1.5% Tween 80, (**D**) 3% Tween 80, (**E**) 1.5% Tween 20, and (**F**) 3% Tween 20.

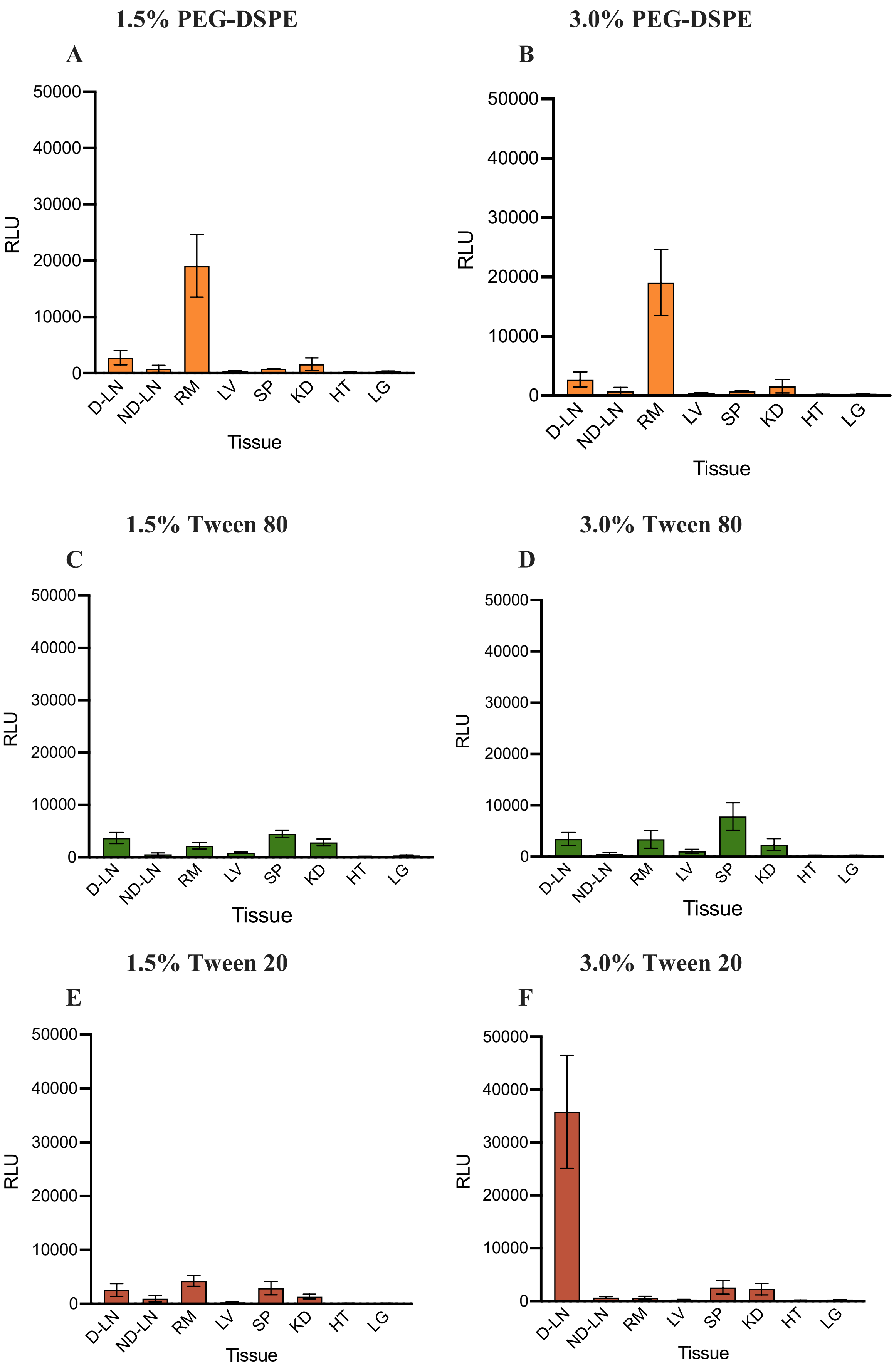

Figure S5. Plate Reader In-vivo data represented as RLU: (**A**) 1.5% PEG-DSPE, (**B**) 3% PEG-DSPE, (**C**) 1.5% Tween 80, (**D**) 3% Tween 80, (**E**) 1.5% Tween 20, and (**F**) 3% Tween 20.

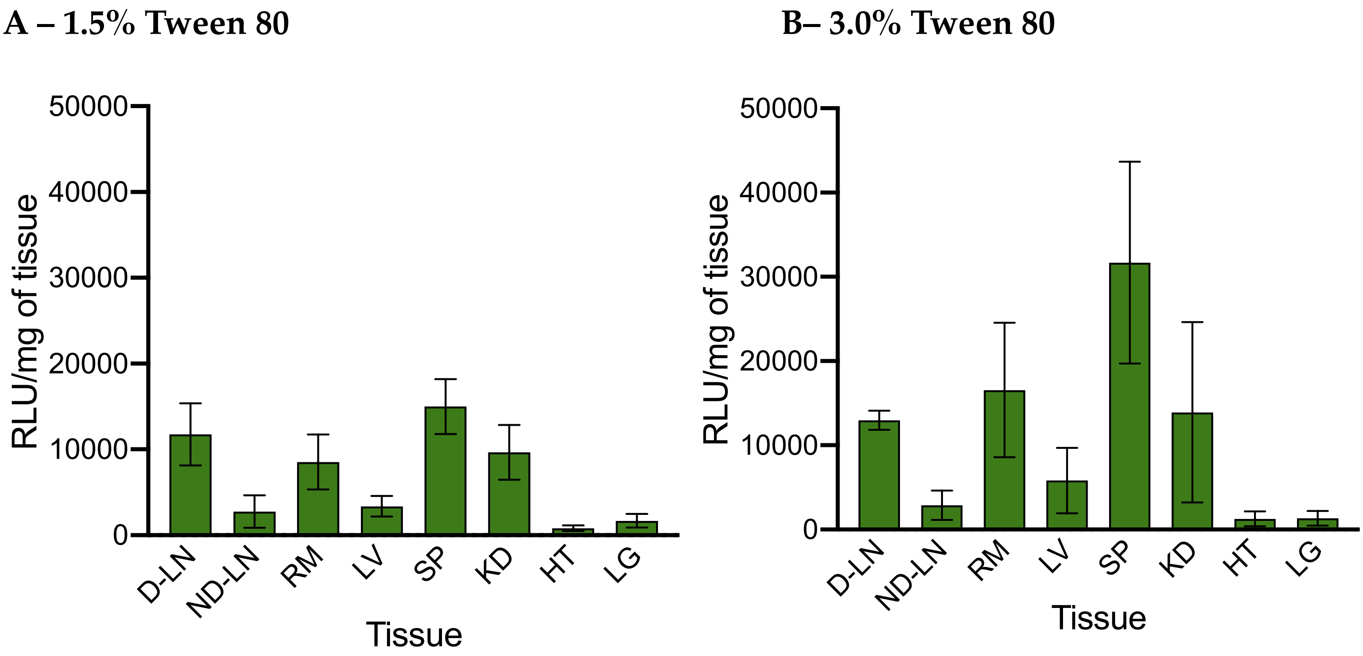

Figure S6. Plate Reader In-vivo data represented as (RLU/mg tissue) for Tween 80 (different scale to Figure 6): (**A**) 1.5% Tween 80 and (**B**) 3% Tween 80.
